# O.5.2-3 Women’s experiences of being physically active during the menopause life stage

**DOI:** 10.1093/eurpub/ckad133.244

**Published:** 2023-09-11

**Authors:** Claire Fitzsimons, Janis Reid, Divya Sivaramakrishnan, Nanette Mutrie, Ailsa Niven

**Affiliations:** Physical Activity for Health Research Centre, The University of Edinburgh, UK; claire.fitzsimons@ed.ac.uk; Physical Activity for Health Research Centre, The University of Edinburgh, UK; claire.fitzsimons@ed.ac.uk; Physical Activity for Health Research Centre, The University of Edinburgh, UK; claire.fitzsimons@ed.ac.uk; Physical Activity for Health Research Centre, The University of Edinburgh, UK; claire.fitzsimons@ed.ac.uk; Physical Activity for Health Research Centre, The University of Edinburgh, UK; claire.fitzsimons@ed.ac.uk

## Abstract

**Purpose:**

There is growing evidence for the benefits of physical activity (PA) during the menopause life stage (MLS) in relation to general health and wellbeing, managing symptoms, and protecting against future ill health. However, Scottish national surveillance data shows a decline in women achieving PA guidelines across the MLS (40% 35-44yrs, 28% 55-64yrs). This work was part of a larger research study. In this abstract, we present qualitative data on women’s experiences of being physically active during the MLS, with a particular interest on mental wellbeing (MWB).

**Methods:**

Four focus groups took place (MS Teams) with women experiencing the MLS (Oct 2022, N = 24, mean age 52.6yrs). Discussion was structured around four questions – what helps/hinders PA during the MLS; how does/did PA influence mental wellbeing during the MLS; and what could be done better/differently to support women to be active during the MLS. Informed by contemporary understanding of behavior, a thematic analysis was undertaken to identify the role of Capability, Opportunity and Motivation on Behaviour (COM-B).

**Results:**

Physical and psychological capability to be active was negatively impacted by menopause symptoms including painful joints, heavy periods, tiredness, and declining mental health. Having access to HRT helped manage menopausal symptoms and therefore increase PA. A lack of time, suitable opportunities, and lack of support from healthcare professionals limited physical opportunities, while the support of other people and social connections enhanced PA. Motivation and confidence to be active was suppressed, but many were aware PA eased psychological symptoms of the MLS, particularly if outdoors. A range of suggestions were made to support PA including enhanced education and opportunities, increased social support, greater access to tools and resources, and prioritising self-care.

**Conclusions:**

Multiple factors influenced PA behaviour during the MLS, and the COM-B model provided a useful framework to understand these. Supporting PA during the MLS is a priority to prevent PA levels falling during this life-stage that then persists into older age. A toolbox of strategies are needed in order to better support women to be active during the MLS.

**Support/Funding Source:**

This work was funded by SAMH, the Scottish Association for Mental Health.

